# Increase in weighting of vision vs. proprioception associated with force field adaptation

**DOI:** 10.1038/s41598-019-46625-7

**Published:** 2019-07-15

**Authors:** Brandon M. Sexton, Yang Liu, Hannah J. Block

**Affiliations:** Department of Kinesiology & Program in Neuroscience, Indiana University Bloomington, Bloomington, USA

**Keywords:** Motor control, Sensorimotor processing, Somatosensory system, Perception

## Abstract

Hand position can be estimated by vision and proprioception (position sense). The brain is thought to weight and integrate these percepts to form a multisensory estimate of hand position with which to guide movement. Force field adaptation, a type of cerebellum-dependent motor learning, is associated with both motor and proprioceptive changes. The cerebellum has connections with multisensory parietal regions; however, it is unknown if force adaptation is associated with changes in multisensory perception. If force adaptation affects all relevant sensory modalities similarly, the brain’s weighting of vision vs. proprioception should be maintained. Alternatively, if force perturbation is interpreted as somatosensory unreliability, vision may be up-weighted relative to proprioception. We assessed visuo-proprioceptive weighting with a perceptual estimation task before and after subjects performed straight-ahead reaches grasping a robotic manipulandum. Each subject performed one session with a clockwise or counter-clockwise velocity-dependent force field, and one session in a null field. Subjects increased their weight of vision vs. proprioception in the force field session relative to the null session, regardless of force field direction, in the straight-ahead dimension (F_1,44_ = 5.13, p = 0.029). This suggests that force field adaptation is associated with an increase in the brain’s weighting of vision vs. proprioception.

## Introduction

To keep voluntary movement accurate in the face of internal or environmental perturbations, the brain may make adjustments in both sensory and motor systems. In the context of motor learning, sensory changes have been suggested in both animal^1^ and human studies^2^. Xerri *et al*. (1999) trained monkeys to pick up food pellets. As the monkeys learned the task, they began using smaller regions of their fingers to pick up the pellets. The corresponding representations in somatosensory cortex (S1) grew about two times larger than the same S1 fingertip regions for the contralateral hand, suggesting an effect of motor learning on the somatosensory system^1^. Ostry *et al*. (2010) instructed human subjects to make straight-ahead reaches while grasping a robotic manipulandum that applied a velocity-dependent force field. Initial movement errors were reduced with trial-and-error practice, a sign of cerebellum-dependent motor adaptation^3–5^; interestingly, Ostry *et al*. (2010) found systematic changes in somatosensation (kinesthesia) in the adapted arm. These perceptual changes may indicate the involvement of somatosensory cortex in motor adaptation^6,7^. In addition, visuomotor adaptation to a cursor rotation results in systematic proprioceptive changes for the adapted hand^8,9^, which is consistent with somatosensory involvement in motor learning whether the perturbation is visual (cursor rotation) or somatosensory (force field).

While somatosensory involvement in motor adaptation has been an important area of investigation in the last ten years, the potential role of multisensory processing in motor adaptation has yet to be considered. Multiple sensory systems play an important role in voluntary movement. For example, to plan an accurate reach, the brain must have an accurate initial estimate of the hand’s position. We normally have access to true hand position (*Y*) through vision and proprioception. The image of the hand on the retina provides a visual estimate (*Ŷ*_*V*_), while receptors in the muscles and joints of the arm provide a proprioceptive estimate (*Ŷ*_*P*_). To form a single estimate with which to guide behavior, the brain is thought to weight and combine them into a single, integrated estimate of hand position^10^. If *Ŷ*_*VP*_ is this integrated estimate, and *W*_*V*_ is the weight of vision vs. proprioception (*W*_*V*_ < 0.5 implies greater reliance on proprioception):

1 $${\hat{{\rm{Y}}}}_{VP}={W}_{V}{\hat{{\rm{Y}}}}_{V}+(1-{W}_{V}){\hat{{\rm{Y}}}}_{P}$$

Although the neural basis of this process is unknown, it may involve multisensory regions of posterior parietal cortex such as the intraparietal sulcus^11^ or angular gyrus^12^. The weighting of each sensory input is thought to be inversely proportional to the associated variance; this is known as the minimum variance or maximum likelihood estimation model^13^, and it has experimental support from a variety of human behaviors^10,14–16^.

Importantly, the reliability of a given sensory input, and its weighting in multisensory integration, is not constant, but may vary with environmental conditions^17^ and locus of attention^18–20^. Thus, multisensory integration can respond to changes in the body or environment that affect sensory perception. For example, a decrease in illumination likely results in a multisensory estimate that relies more on proprioception than vision^17^. In addition, the computation being performed may affect sensory weighting; subjects rely more on vision for planning movement vectors and more on proprioception for planning the joint-based motor command^21^. The modality of the target being reached also plays a role, with subjects minimizing coordinate transformations by relying more on vision when reaching to visual targets, and more on proprioception when reaching to proprioceptive targets^22^. Even different aspects of what might be considered the same computation can use different weightings; vision is weighted more heavily relative to proprioception when localizing the hand in azimuth than in depth^23^.

Most of the evidence of multisensory involvement in motor learning comes from studies asking which sensory signals are necessary for force field adaptation. This form of motor adaptation can occur without a proprioceptive error, using visual feedback^24–26^, but also without a visual error, using only proprioception^25,27^. This suggests subjects can flexibly use available error information, whether visual or proprioceptive^25^. To our knowledge, only one study has considered force field effects on vision and proprioception in the same experiment: Haith *et al*. (2008) had subjects point at a series of visual or proprioceptive targets interspersed with force field adaptation trials. Results suggest spatial recalibration of both visual and proprioceptive estimates occurs after force field learning^28^. However, simultaneous visual and proprioceptive processing, where visuo-proprioceptive integration can occur, has not been considered in the context of force adaptation. Multisensory integration, unlike intersensory interactions, can only be assessed by looking at what subjects do when multiple modalities are available at the same time and integration is thus possible. In other words, to test whether force field adaptation affects the weighting and combining of visual and proprioceptive information to create an integrated multisensory estimate of hand position, sensory estimation trials with simultaneous visual and proprioceptive information about hand position would be required.

Here we asked whether force field adaptation affects the brain’s weighting of visual and proprioceptive estimates of hand position when both are available. Given that multisensory integration plays a key role in movement planning, one possibility is that force field adaptation affects all relevant sensory modalities similarly, such that a constant weighting of vision vs. proprioception is maintained. Alternatively, the somatosensory perturbation could be considered a source of proprioceptive unreliability, which we would expect to result in vision being up-weighted relative to proprioception. We assessed visuo-proprioceptive weighting with a perceptual estimation task before and after subjects performed straight-ahead reaches while grasping a robotic manipulandum. Each subject performed one session with reaches in a clockwise or counter-clockwise velocity-dependent force field, and one session in a null field to control for perceptual changes not specific to force adaptation.

## Methods

### Subjects

46 healthy right-handed adults (aged 18–33, mean age 22.7 years; 22 female) completed two sessions each, scheduled at least 4 days apart. We can roughly estimate from the standard deviation of weighting changes in an earlier experiment using a similar sensory estimation task^18^ that to detect a 10% change in weight of vision vs. proprioception with 90% power (α = 0.05), we would need a sample size of 21 per group. Subjects reported that they were free of neurological or musculoskeletal problems, and had normal or corrected-to-normal vision in both eyes. All procedures were approved by the Indiana University Institutional Review Board and carried out in accordance with IRB guidelines and regulations. All subjects provided written informed consent.

### Session design

Subjects were randomly assigned to the clockwise (CW) or counterclockwise (CCW) group (N = 23 each), and to have the real or null session first. In each session, subjects performed a series of straight-ahead reaching movements grasping a robotic manipulandum (KINARM End Point, BKIN), with a sensory estimation task to assess visual and proprioceptive perception of hand position before and after the reaching task. In the real session, subjects adapted to a CW or CCW velocity-dependent force field, according to group assignment. The null session comprised the same number of reaching movements, but no force field. This was intended to control for any perceptual changes not specific to force field adaptation, such as making reaching movements, grasping the manipulandum, viewing the task display, etc.

**Figure 1:**
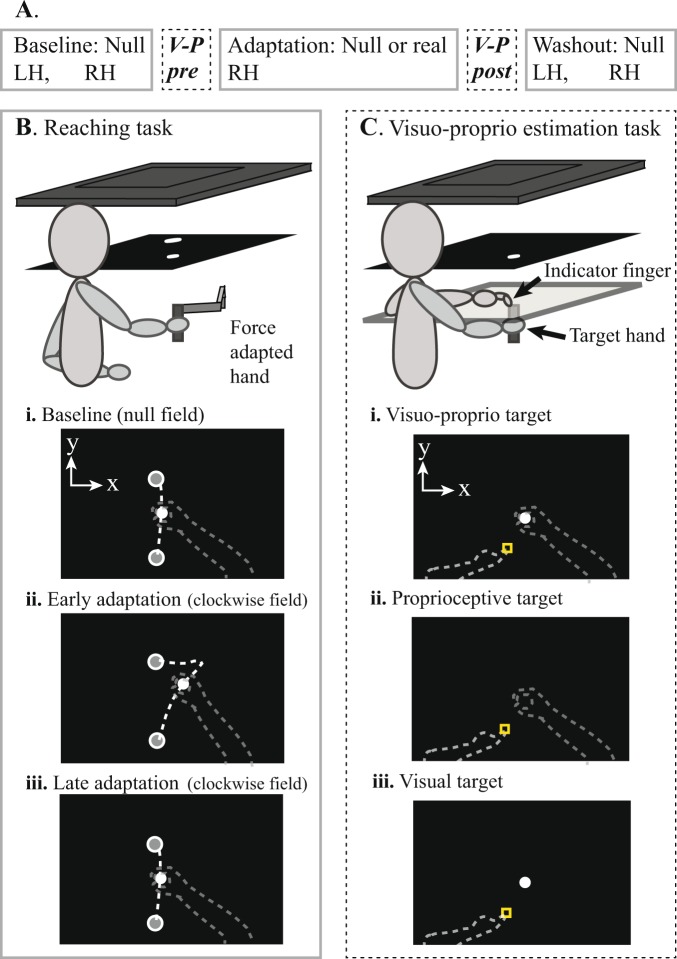
(**A**) Single session protocol. After practicing each task, subjects completed five blocks of reaching (solid outline) and two blocks of visual and proprioceptive estimates (dashed outline). Each subject performed two sessions, one of which included a velocity-dependent force field during the adaptation block. Subjects had no direct vision of either hand at any point in the session. (**B**) Reaching task. Subjects viewed images displayed on a TV (top) in a horizontal mirror. Subjects were instructed to make a series of straight-ahead movements grasping a robotic manipulandum. Images in the mirror appeared to be in the plane of the manipulandum, which was represented continuously by a white cursor. (**i**) Top-down view of the display. Dashed lines not visible to subject. During baseline and washout blocks of both sessions, and the adaptation block of the null session, subjects moved the passive manipulandum (null field) from the start circle to the target circle, both at body midline. (**ii**) A CW or CCW field was applied during the adaptation block of the real session only. Reaching paths early in this block had large rightward or leftward perpendicular deviations, respectively. (**iii**) After subjects had adapted to the force field, reaching trajectories became straight, with perpendicular deviations similar to baseline. (**C**) Visuo-proprioceptive estimation task. At the same apparatus, a touchscreen was placed directly over the manipulandum. Subjects moved their left (indicator) index finger, on top of the touchscreen, from a variable start position (square) to the perceived location of targets related to the right hand (target hand), which was below the touchscreen. Subjects were instructed to take their time and place their indicator finger as accurately as possible. No online or endpoint feedback about the indicator finger was given. (**i**) For the VP target, a white disc appeared above the center of the manipulandum handle, grasped in the right hand. (**ii**) The P target was the same except no white disc was displayed. (**iii**) The V target was the white disc alone, with the right hand resting in the subject’s lap.

Each session consisted of five blocks of trials (Fig. 1A): baseline reaching in the null field with the left and right hands (16 trials each hand); pre-adaptation visuo-proprioceptive estimation task (35 trials); adaptation block of reaching with the right hand (208 trials with the right hand in a real or null field depending on session); post-adaptation visuo-proprioceptive estimation task (35 trials); and washout reaching in the null field with the left and right hands (16 trials each hand). Left hand baseline and washout reaching trials were included so we could assess any intermanual transfer of force field learning, as the left hand was used to indicate the subject’s perception of right hand position in the visuo-proprioceptive estimation task.

### Apparatus

Subjects were seated in front of a reflected rear projection apparatus (TV and mirror) throughout the session, such that the task display appeared to be in the same horizontal plane as the manipulandum (Fig. 1). Both hands remained below the mirror at all times, preventing direct vision of either hand, and a cape attached to the edge of the mirror was draped over subjects’ shoulders to prevent vision of the upper arms. Subjects were centered with the apparatus and strapped to the chair with a harness to limit torso motion. A headband across the forehead was attached to the edge of the TV with Velcro, restricting subjects’ head motion.

### Reaching task

Subjects grasped the manipulandum handle and made a series of straight-ahead movements to a visual target 20 cm from the start position (Fig. 1B). A 1 cm white disc was displayed over the center of the manipulandum handle, providing online feedback throughout the reach. Subjects were instructed to make their movement paths straight and brisk. During the adaptation block of the real force session only, subjects experienced a CW or CCW velocity-dependent force field (Fig. 1Bii):

2 $$[\begin{array}{c}{f}_{x}\\ {f}_{y}\end{array}]=D[\begin{array}{cc}0 & 18\\ -18 & 0\end{array}][\begin{array}{c}{v}_{x}\\ {v}_{y}\end{array}]$$

where *f*_*x*_ and *f*_*y*_ are the commanded force to the manipulandum in the lateral (x) and sagittal (y) directions, *v*_*x*_ and *v*_*y*_ are hand velocities, and *D* is the force direction (1 for CW, −1 for CCW). The desired movement time was 575–650 ms, and subjects received feedback telling them when movements were too fast or too slow. Each trial ended when the manipulandum reached the target, at which point the hand was passively moved back to the start position. The maximum perpendicular deviation of the manipulandum from a straight-line path was computed for each trial as a measure of movement error. To normalize movement errors to baseline levels, max perpendicular deviation at the end of right hand baseline was subtracted from every right hand trial, and max perpendicular deviation at the end of left hand baseline was subtracted from every left hand trial.

To quantify the degree of perturbation at the beginning of the adaptation block, we averaged max perpendicular error on the first 8 trials of adaptation. The washout blocks were used to estimate negative aftereffect (1) in the right hand as a measure of force field learning, and (2) in the left hand to measure transfer of learning to the untrained left hand, which was used to indicate the subject’s perceptions in the sensory estimation task. Negative aftereffect was estimated by taking the mean of the first 8 washout trials in each hand.

### Visuo-proprioceptive estimation task

Immediately before and after the force adaptation block, subjects performed a sensory estimation task to assess visual and proprioceptive estimates of their right hand position (Fig. 1C). With no direct vision of either hand, subjects used their left (indicator) index finger to point to the perceived location of three target types^15,18,29,30^: a visuo-proprioceptive target (1 cm white disc displayed directly above their right hand, which grasped a stationary manipulandum handle beneath the touchscreen glass) (Fig. 1Ci); proprioceptive-only target (right hand grasping the handle, with no white disc) (Fig. 1Cii); and a visual-only target (white disc alone, with the right hand lowered to rest in the subject’s lap) (Fig. 1Ciii). For this task, a touchscreen, consisting of a 3-mm pane of glass with an infrared touch overlay (PQ Labs), was slid into place directly above the robotic manipulandum (and below the mirror) to record indicator finger positions. To prevent subjects from learning or repeating a particular movement direction or extent with the left hand, indicator finger start position was randomized trial-to-trial between five start positions, and targets between two target positions, all centered with the body midline. Subjects received step-by-step instructions to guide them through the task via pre-recorded audio prompts. Subjects received no online or endpoint feedback about the left indicator finger and no knowledge of results. There were no speed requirements, and subjects were instructed to take their time and be as accurate as possible. Subjects were asked not to slide their finger on the glass. Adjustments of indicator finger position were permitted, with the final position recorded once the finger had not moved more than 2 mm in 2 seconds.

**Figure 2:**
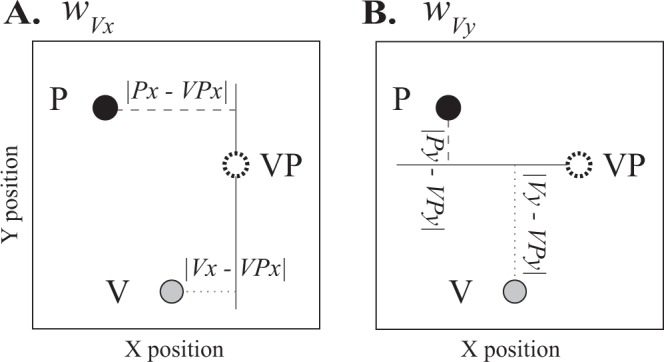
Computing weight of vision vs. proprioception. In this schematic diagram, the subject’s estimate of V, P, and VP target positions are represented by a grey, black, and white disc, respectively. (**A**) *w*_*Vx*_ is computed by dividing the P-to-VP distance in the x-dimension (dashed line) by the sum of the P-to-VP distance and the V-to-VP distance in the x-dimension (dashed and dotted lines). (**B**) *w*_*Vy*_ is computed similarly, with all distances in the y-dimension.

Each block of the visuo-proprioceptive estimation task (pre- and post-adaptation) comprised 35 trials: 15 visual-only (V), 15 proprioceptive-only (P), and 5 visuo-proprioceptive (VP) trials, in pseudorandom order. We computed an estimate of subjects’ weighting of vision vs. proprioception (*w*_*V*_) when both modalities were available, on VP targets^18^. We computed *w*_*V*_ separately in the lateral (*w*_*Vx*_) and sagittal dimension (*w*_*Vy*_) because *w*_*V*_ has been observed to differ across spatial dimensions^13,16,23^. van Beers *et al*. (2002) suggested the brain determines lateral and sagittal weights independently, based on differences in the spatial properties of visual and proprioceptive variance. To compute *w*_*V*_ in each dimension, we divided the distance between P and VP target estimates by the sum of the P-to-VP and V-to-VP distances. For the lateral (x) dimension (Fig. 2A):

3 $${w}_{Vx}=\frac{|Px-VPx|}{|Px-VPx|+|Vx-VPx|}$$

where *|Px* − *VPx|* and *|Vx* − *VPx|* are the x-dimension distances between the mean final position of the indicator finger on P or V targets, respectively, and the mean position of the indicator finger on VP targets. Similarly, for the sagittal (y) dimension (Fig. 2B):

4 $${w}_{Vy}=\frac{|Py-VPy|}{|Py-VPy|+|Vy-VPy|}$$

In other words, if VP endpoint positions are closer to P than V positions in the y-dimension, the subject relied more on proprioception than vision (*w*_*Vy*_ < 0.5). This method takes advantage of the different spatial biases inherent in vision and proprioception^31,32^, even with no perturbation^33^. Because *w*_*V*_ undergoes small fluctuations over time, as we have done previously^18^, we computed a separate *w*_*V*_ for each of the 5 VP trials pre- and post-adaptation, comparing each VP trial indicator finger endpoint with the means of the 4 V and 4 P trials occurring closest in time. The 5 values were then averaged for each subject to give a weighting estimate for the pre- or post-adaptation sensory task.

It is important to note that this method does not rely on variance or precision of the endpoints for the different target types, only spatial bias. Weights based on endpoint precision are a predicted weight, usually assuming the subject follows minimum variance integration^16,34^. Subjects’ weight of vision vs. proprioception can also be inferred from their adaptation^23^ or, as we have done here, from their spatial biases in estimating V, P, and VP targets^12,18,29,35^. This latter method does not involve the precision or scatter of the endpoints, so the number of trials is less critical. Any effect of force field adaptation on *w*_*V*_ could potentially dissipate in a few minutes, given that *w*_*V*_ can quickly change based on the subject’s attention and environment^18^. Therefore, because we were not trying to generate variance-based predictions of *w*_*V*_, we chose the spatial bias method to reduce the number of trials. The spatial estimation task incorporates many pauses and audio prompts compared to reaching with the robotic manipulandum, so 35 trials (15 V, 15 P, 5 VP) took most subjects about 10 minutes.

### Statistical analysis

All statistical inferences were performed two-tailed, with α of 0.05.

The two primary outcome variables were *w*_*Vx*_ and *w*_*Vy*._ A separate mixed model 2 × 2 × 2 ANOVA (timepoint x session x group) was used to analyze each of these. Timepoint (pre- and post-adaptation) and session (real and null) were within-subjects factors, and group (CW and CCW force field) was a between-subjects factor. A significant 3-way interaction (timepoint x session x group) would indicate that the variable changed differently in the real vs. null sessions, *and* that force field direction matters. In the absence of the 3-way interaction, a significant timepoint x session interaction would indicate that the variable changed differently in the real vs. null session, but force field direction (i.e., group) does not make a difference. Because each ANOVA was 2 × 2 × 2, no post-hoc pairwise comparisons were necessary. Consistent with the idea that weights in the sagittal and lateral dimensions are determined independently^23^, *w*_*Vx*_ and *w*_*Vy*_ were not correlated with each other (r = 0.07).

To evaluate whether our sensory estimation task could replicate findings of spatial shifts in proprioceptive or visual estimate, we also analyzed Px, Py, Vx, and Vy as secondary outcome variables. We performed a mixed model 2 × 2 × 2 ANOVA on each of these, with the same factors as the *w*_*V*_ ANOVAs. The remaining secondary outcome variables consisted of force field adaptation and aftereffect magnitudes. To evaluate whether either hand experienced aftereffects after force field adaptation, we performed a separate paired-sample t-test for each hand in each group. In each case, we compared max perpendicular error in the appropriate washout block (right or left hand) across the real and null sessions. A significant difference between sessions would suggest the presence of aftereffect for that hand. We predicted aftereffects in the right hand, which was exposed to the force field, but not in the left hand, which was not exposed. Because this research is exploratory, we did not adjust p-values to compensate for analyzing multiple outcome variables^36^. Therefore, statistically significant results should be regarded as an indication for further study, rather than as confirmatory.

## Results

All means are given with their 95% confidence intervals (CI).

### Reaching task

**Figure 3:**
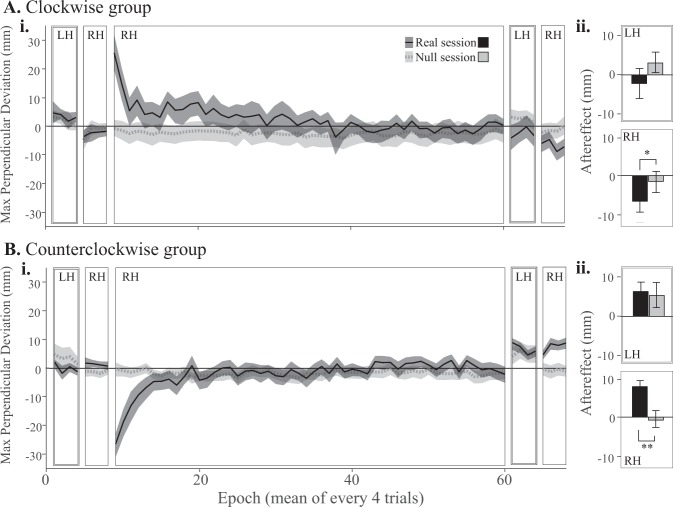
Reaching errors for the clockwise (**A**) and counterclockwise (**B**) groups. Positive values reflect rightward errors. (**i**) Max perpendicular reaching error in epochs of 4 trials, averaged across subjects with standard errors (shaded regions). In the real session (black solid line) subjects experienced a CW or CCW force field with the right hand, after a short baseline with each hand in the null field. Initial large errors on exposure to the force field decreased to near-baseline levels by the end of the adaptation block. Finally, washout blocks in the null field were used to assess negative aftereffects of force field adaptation in each hand. (**ii**) Mean aftereffect in each hand with standard errors. Top: In the left hand, neither group showed a significant difference between real and null sessions, suggesting little or no transfer of learning to the left hand in the real session. Bottom: In the right hand, both groups showed some evidence of an aftereffect in the real relative to null session, suggesting that force field adaptation occurred robustly in the real session. *p < 0.1. **p < 0.05.

In the real session, both groups had small perpendicular errors during baseline. Both CW and CCW groups initially had large perpendicular errors when the force was introduced: 20 ± 9 mm rightward and 23 ± 8 mm leftward, respectively (mean ± 95% CI). In contrast, at the same point in the null session, error was only 1 ± 5 mm leftward for the CW group, and 0.5 ± 4 mm leftward for the CCW group. After 208 force field trials in the real session’s adaptation block, error returned to approximately baseline levels, suggesting adaptation to the perturbation had occurred (Fig. 3). In the null session, max perpendicular errors were small throughout the reaching task for both groups, as expected, although blocks performed with the left hand appeared to have slight rightward errors compared to blocks performed with the right hand (Fig. 3).

Post-adaptation washout blocks performed with the right hand tended to have larger perpendicular errors in the real session than the null session—leftward for the CW group and rightward for the CCW group—suggesting the presence of negative aftereffect. This difference did not reach statistical significance for the CW group (t_22_ = −1.98, p = 0.06), but did for the CCW group (t_22_ = 2.69, p = 0.013). In the CW group, right hand aftereffect was 7 ± 5 mm to the left in the real session, and 1 ± 5 mm to the left in the null session (Fig. 3A). In the CCW group, right hand aftereffect was 8 ± 3 mm to the right in the real session, and 0.6 ± 4 mm to the left in the null session (Fig. 3B). These adaptation and aftereffect magnitudes are similar to those that have been observed by others with a similar paradigm^37^.

Post-adaptation washout blocks performed with the left hand were similar in the real and null sessions (Fig. 3), which is not consistent with intermanual transfer of learning. In the CW group, left hand aftereffect was 2 ± 7 mm to the left in the real session, and 3 ± 5 mm to the right in the null session (t_22_ = −1.02, p = 0.32). In the CCW group, left hand aftereffect was 6 ± 4 mm to the right in the real session, and 5 ± 6 mm to the right in the null session (t_22_ = 0.27, p = 0.79).

### Weighting of vision vs. proprioception (*w*_*V*_)

**Figure 4:**
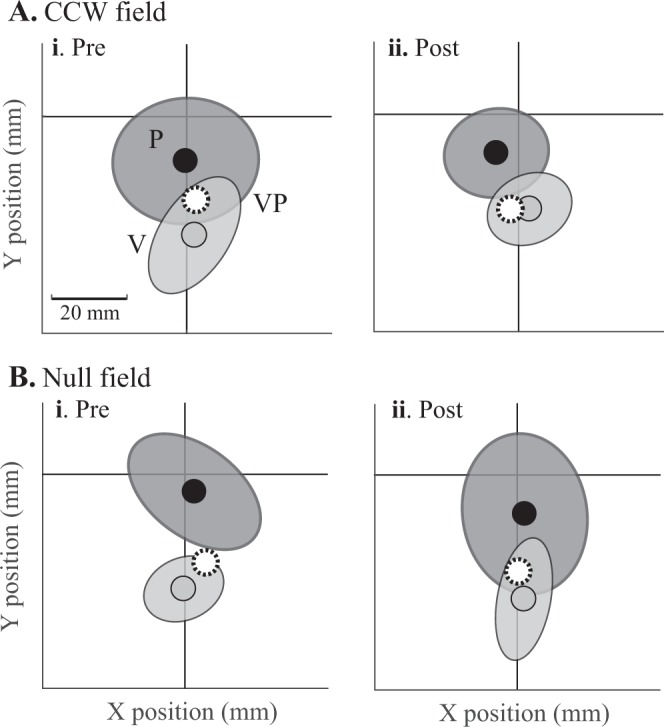
Visuo-proprioceptive estimation data from a subject in the CCW group. Subject was seated in the direction of the negative y-axis, with the target always at the origin. Mean estimates of VP, V, and P targets, with standard error ellipses for the latter two target types. Weight of vision relative to proprioception (*w*_*V*_) is computed by comparing relative spatial biases in the three estimates. (**A**) Real session. (**i**) Before adapting to the CCW force field, the VP target is estimated midway between the V and P targets in the y-dimension, indicating relatively equal reliance on vision and proprioception (*w*_*Vy*_ = 0.53). (**ii**) After adapting to the force field, the VP target is estimated closer to the V than the P target in the y-dimension, indicating greater reliance on vision (*w*_*Vy*_ = 0.99). In contrast, x-dimension weighting decreased slightly in this session (*w*_*Vx*_ = 0.57 pre-adaptation and 0.50 post-adaptation). (**B**) Null session. On another day, *w*_*Vy*_ decreased slightly after an equal number of reaching trials in a null field (0.73 pre and 0.68 post), and *w*_*Vx*_ increased (0.48 pre and 0.62 post).

**Figure 5:**
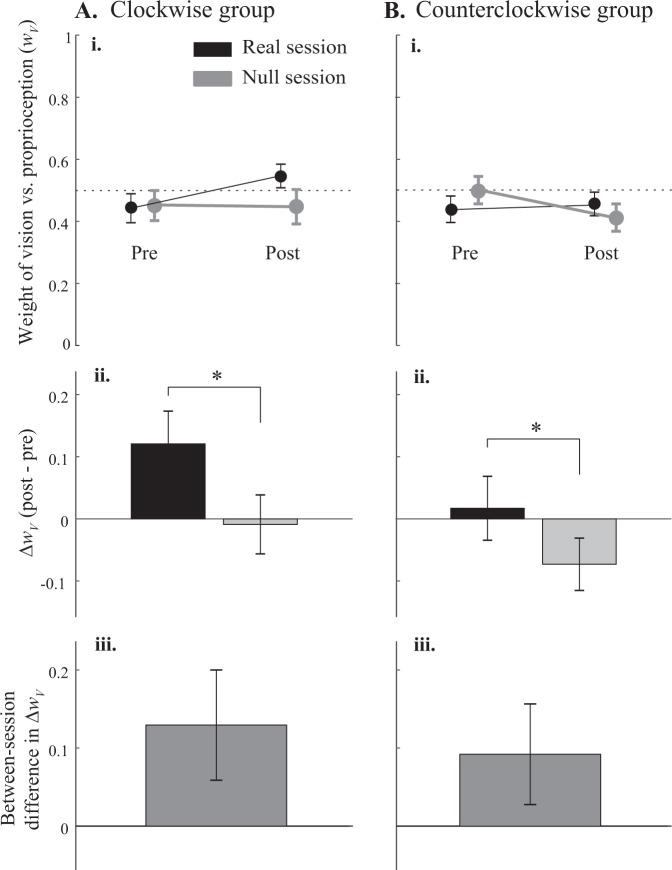
Weight of vision versus proprioception in the sagittal dimension (*w*_*Vy*_) for the clockwise (**A**) and counterclockwise (**B**) groups. (**i**) Mean *w*_*Vy*_ pre- and post-adaptation block in the real (black) and null (grey) session. 0 corresponds to total reliance on proprioception, and 1 corresponds to total reliance on vision. (**ii**) Mean within-session change in *w*_*Vy*_. Positive values indicate increased reliance on vision, while negative values indicate increased reliance on proprioception. *A significant interaction between session (real, null) and timepoint (pre, post) suggests that *w*_*Vy*_ increased after real, but not null, force adaptation, whether the force field was clockwise or counterclockwise. (**iii**) Mean between-session difference in Δ*w*_*Vy*_. All error bars represent standard errors.

In the visuo-proprioceptive estimation task, the example subject in Fig. 4 increased their reliance on vision over proprioception in the y-dimension (positive Δ*w*_*Vy*_) in the real (CCW field) session, but not the null session. In contrast, Δ*w*_*Vx*_ was slightly negative in the real session, but positive in the null session (Fig. 4). At the group level, we found Δ*w*_*Vy*_ to be consistently more positive in the real session than the null session, for both groups (Fig. 5 and Supplementary Figs S1 and S2). In the CW group, Δ*w*_*Vy*_ was 0.12 ± 0.10 for the real session (mean ± 95% CI) and 0.006 ± 0.094 for the null session. In the CCW group, Δ*w*_*Vy*_ was 0.020 ± 0.10 for the real session and −0.075 ± 0.083 for the null session. There was no main effect of timepoint (pre, post), session (real, null) or group (CW, CCW) for *w*_*Vy*_ (F_1,44_ = 0.54, 0.49, 0.32, respectively, and p = 0.46, 0.49, and 0.57, respectively). However, there was a significant interaction of timepoint × session (F_1,44_ = 5.13, p = 0.029). The interaction of timepoint × session × group was not significant (F_1,44_ = 0.08, p = 0.78), nor was the interaction of session x group (F_1,44_ = 1.0, p = 0.32). The interaction of timepoint × group did not reach significance (F_1,44_ = 3.61, p = 0.064). Taken together, these results suggest that *w*_*Vy*_ increased more in the real session than the null session (timepoint × session interaction), and this occurred for both the CW and CCW group, as indicated by the lack of timepoint × session × group interaction.

**Figure 6:**
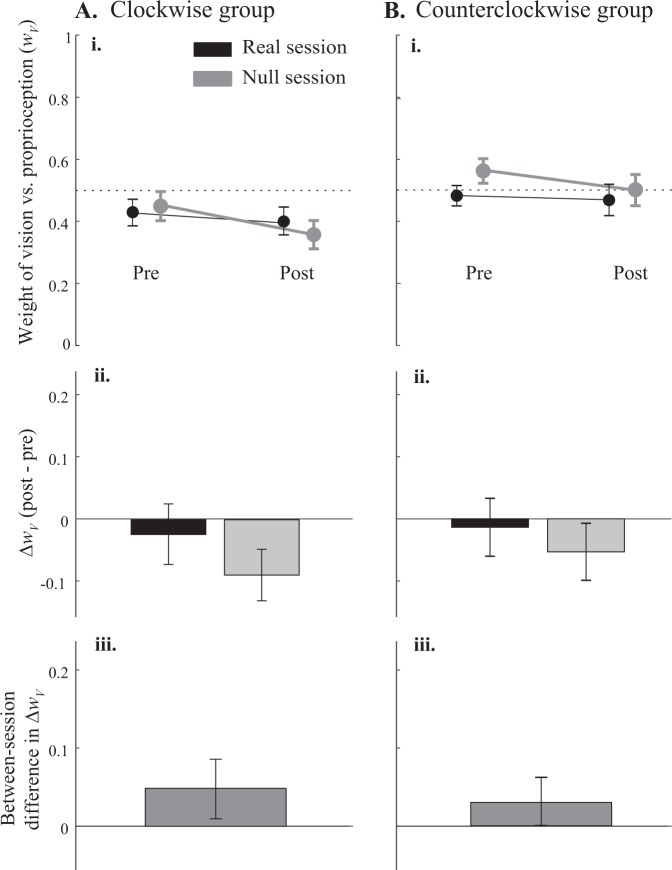
Weight of vision versus proprioception in the lateral dimension (*w*_*Vx*_) for the clockwise (**A**) and counterclockwise (**B**) groups. (**i**) Mean *w*_*Vx*_ pre- and post-adaptation block in the real (black) and null (grey) session. 0 corresponds to total reliance on proprioception, and 1 corresponds to total reliance on vision. (**ii**) Mean within-session change in *w*_*Vx*_. Positive values indicate increased reliance on vision, while negative values indicate increased reliance on proprioception. (**iii**) Mean between-session difference in Δ*w*_*Vx*_. The absence of interaction effects suggests that force adaptation did not significantly affect *w*_*V*_ in the x-dimension. All error bars represent standard errors.

Weighting changes in the x-dimension (Fig. 6) were less consistent than in the y-dimension. In the CW group, Δ*w*_*Vx*_ was −0.031 ± 0.093 for the real session (mean ± 95% CI) and −0.088 ± 0.079 for the null session. In the CCW group, Δ*w*_*Vx*_ was −0.014 ± 0.089 for the real session and −0.049 ± 0.090 for the null session. In other words, on average, subjects reduced their reliance on vision and increased their reliance on proprioception in the x-dimension, regardless of session or group (main effect of timepoint, F_1,44_ = 4.34, p = 0.043). There was no effect of session (F_1,44_ = 1.0, p = 0.32) and no significant interactions (all p > 0.2). However, the groups apparently differed in some way on *w*_*Vx*_ (main effect of group, F_1,44_ = 5.82, p = 0.020). Because we have previously observed that subjects vary substantially in weighting of vision vs. proprioception even with no perturbation, we wondered if pre-adaptation *w*_*Vx*_ in either of our two groups differed substantially from subjects in earlier studies. To find out, we compared pre-adaptation *w*_*Vx*_ in the CW and CCW group with *w*_*Vx*_ in 80 healthy young adults (mean age 23.4 years) described previously^15^. Mean *w*_*Vx*_ in these three samples was 0.43 ± 0.07, 0.53 ± 0.05, and 0.49 ± 0.04 (mean ± 95% CI), respectively. A one-way ANOVA found no significant differences among these three samples (F_2,125_ = 1.71, p = 0.18). In other words, although pre-adaptation *w*_*Vx*_ favored proprioception more in the CW group than in the CCW group, perhaps explaining the main effect of group on *w*_*Vx*_ in the present study, neither sample differed significantly from subjects we have tested previously.

### Spatial shifts in visual and proprioceptive estimates

We did not observe any pattern of shifts in visual or proprioceptive estimates that would be consistent with previous literature^28,38^. Specifically, if either estimate had shifted laterally in a direction related to force field direction, we would have predicted a significant timepoint × session × group interaction. However, there was no such interaction for Px (F_1,44_ = 0.022, p = 0.88; Supplementary Fig. S3) or Vx (F_1,44_ = 0.028, p = 0.87, Supplementary Fig. S4). Since we observed the greatest change in *w*_*v*_ in the sagittal dimension, we also looked at Py and Vy, but neither showed the three-way interaction (F_1,44_ = 0.21, 140, and p = 0.65, 0.24, respectively; Supplementary Figs S5 and S6).

## Discussion

Here we asked whether force field adaptation is associated with changes in visuo-proprioceptive weighting. Subjects increased their weight of vision vs. proprioception in the force field session relative to the null field session, regardless of force field direction, in the straight-ahead dimension. This increase in reliance on vision over proprioception could indicate that the brain interprets the force field as a somatosensory perturbation or sign of proprioceptive unreliability.

### Multisensory processing in motor control

Inherent in any target-directed hand movement is an estimate of hand position, which can be encoded by both vision and proprioception. The brain is thought to weight and combine available sensory estimates to form an integrated multisensory estimate of hand position with which to guide movement^10^. Multisensory research has made substantial progress in determining the principles by which multisensory integration occurs and demonstrating their relevance in human perception^39,40^. In addition, it is known that multisensory weights have a role in motor control. E.g., different visuo-proprioceptive weights are evident for different reach target modalities and reach directions^22^. However, it is not clear whether a perceptual computation such as visuo-proprioceptive weighting is affected by, or plays a role in, motor adaptation or learning.

Motor adaptation is a trial-by-trial process of reducing movement error. E.g., visuomotor adaptation occurs in response to a visual perturbation. A visuomotor rotation paradigm deviates the cursor representing hand position by some angular offset such as 30°. With practice, subjects adapt their movements so the cursor reaches the target. Shifts in proprioceptive estimates of hand position (proprioceptive realignment or recalibration) have been observed in conjunction with visuomotor adaptation^8,41^, but differing in time course and generalization pattern from visuomotor adaptation^42^. Force field adaptation involves a somatosensory perturbation rather than visual. When forces are systematically applied to the hand during a reach, e.g. pushing the hand to the right when the subject is trying to reach straight ahead, rightward errors occur at first. However, error feedback recalibrates the sensorimotor map, gradually reducing movement error. Until recently, a force perturbation had been thought to elicit motor adaptation only, as visuo-proprioceptive signals remain veridical^41,43^; sensory realignment was thought to require inter-sensory misalignment^44^. However, Ostry *et al*. (2010) tested arm proprioception and found systematic changes during force adaptation, independent of motor adaptation rate^45^. This could reflect a common sensorimotor map, with modifications affecting parameters of multisensory integration.

### Visuo-proprioceptive weighting increased during force field adaptation

To our knowledge, subjects’ weighting of vision vs. proprioception, with both signals available simultaneously, has not been examined in either visuomotor or force field adaptation. We chose to study visuo-proprioceptive weighting in the context of force field adaptation because from the perspective of multisensory processing, the predictions are straightforward: The target is visual, the hand is represented by a visual cursor, and the perturbation disrupting the movement to the target is somatosensory. All these factors would be expected to favor vision over proprioception in the weighting computation. To control for aspects of the task other than the somatosensory perturbation, subjects in the present study performed one session entirely in the null field, and one session with the force perturbation. We predicted that compared to the control session, subjects would up-weight vision relative to proprioception. Our results provide evidence that this is indeed the case. For y-dimension weighting, the presence of a timepoint × session interaction in the absence of a timepoint × session × group interaction indicates that subjects’ increase in weighting of vision was specific to the real force field session, regardless of whether that field was CW or CCW. Thus, unlike force field-related spatial recalibration of proprioception^45,46^ or vision^28^, force field *direction* does not appear to impact visuo-proprioceptive weighting. In other words, people increased their reliance on vision in the sagittal dimension by about 10% in their real session (whether force field was CW or CCW) vs. their null session. That this occurred in both groups’ real but not null sessions suggests it is a robust, though small, effect. To put this in context, we have previously seen a ~8% change in *w*_*V*_ by manipulating error history and a ~20% change by manipulating target salience^18^. The present result may indicate that the force field in general is interpreted by the brain as somatosensory unreliability or perturbation. Literature from postural control supports this conclusion: Decreasing the reliability of somatosensory input increases reliance on the visual and vestibular systems^47^.

It is important to note that we cannot infer causal relationships from these results. It is possible that force field adaptation, or even exposure to the force field, caused subjects to increase their reliance on vision. However, it is also possible that the observed weighting changes form an unrecognized component of motor adaptation whose absence would impair performance. To distinguish these possibilities, future studies could assess motor adaptation rate, magnitude, and retention after manipulating subjects’ visuo-proprioceptive weighting, perhaps via target modality^21^ or salience^18^.

### Results were dimension-specific

Interestingly, we found robust evidence of force field-related increase in visuo-proprioceptive weighting for the y-dimension (sagittal plane), but not the x-dimension (lateral plane). This is somewhat counter-intuitive, given that previous measurements of proprioceptive change have shown effects in the lateral dimension^37,46^. However, the force field used here was a curl field, so it acts in both dimensions. To our knowledge, no one has actually looked for visual or proprioceptive shifts in the sagittal dimension in a forward-reaching task with a curl field, but our results suggest that multiple dimensions should be examined when possible. Haith *et al*.^28^ found significant changes in both vision and proprioception in the lateral but not the sagittal dimension, although they used a lateral field rather than a curl field.

We analyzed the x- and y-dimensions separately because of evidence that visuo-proprioceptive weighting computations vary by spatial dimension^23^. Certain spatial aspects of motor adaptation are also thought to be controlled separately: Distinct coordinate systems for adaptation of movement direction and extent have been observed^48^. The force field perturbation in the present study was a curl field, with equal components in the x- and y-dimensions (eq. 2). However, the desired movement was entirely in the y-dimension, since the target was straight ahead of the starting position. In addition, subjects were explicitly instructed to move the manipulandum handle straight ahead. Since visuo-proprioceptive weighting is affected by locus of attention and target salience^18^, these factors in the task design could have created a situation where the straight-ahead dimension was more sensitive to changes in visuo-proprioceptive weighting associated with the force field. This could be tested by altering the dimensional parameters in a new experiment.

An alternative explanation for the lack of force field session-specific change in weighting for the x-dimension could be the weighting characteristics of the two groups. The main effect of group in this parameter may indicate that subjects in the two groups differed in their x-dimension weighting in general, not in a way that changed differently over time or in different sessions. We have observed substantial inter-subject variability in visuo-proprioceptive weighting even in unperturbed situations^15,18^. However, the absence of a main effect of session for x-dimension weighting, or any interaction involving session, leads us to hypothesize that even two groups with more similar baseline x-dimension weighting would not change differently in the force field relative to null field session.

One result we did not expect was the near-significant interaction of timepoint x group in the y-dimension. It is indeed possible that this variable changed differently in the two groups. However, any such change would have applied similarly in the force and null sessions. In other words, this interaction, even if truly present, was unrelated to the presence of the force field.

### Considerations for a bimanual sensory estimation task

While similar sensorimotor studies have used this method^28,46^, psychometric procedures using two-alternative forced choice (2AFC) tasks are perhaps more common. However, such tasks in motor control experiments have almost always been used to measure proprioceptive spatial alignment, not visuo-proprioceptive integration parameters. A bimanual estimation task presents several advantages over single-modality blocks of 2AFC trials when the goal is to estimate visuo-proprioceptive weighting. Using the left hand, which is not exposed to the force perturbation, to indicate perceived right hand position allows simultaneous estimation in both lateral and sagittal dimensions^49^. It is also more analogous to using sensory information for motor planning, and lends itself to mixing up the visual, proprioceptive, and visuo-proprioceptive trial types to make it apparent that the visual and proprioceptive signals, while sometimes presented alone and sometimes together, relate to the same object. Given that multisensory weighting can change instantaneously and differs for different computations, the bimanual approach is better able to assess the computation of interest, multisensory weighting.

A bimanual task in this situation does carry risks. Namely, any intermanual transfer of force field adaptation to the left hand would bias the sensory estimates. However, this risk is small considering that proprioceptive recalibration^9^ and motor adaptation do not transfer well to movements with different kinematics or contexts^50^, and the movements of the left hand in the sensory task differ in posture, orientation, and movement path from the reaching task. We can reasonably expect that if any transfer were to occur, it should be very small. If there was interlimb transfer of motor or sensory changes to the left hand, then we would have predicted negative aftereffects in the left hand. We tested for such aftereffects by including left hand reaches in the null field pre- and post-adaptation block. However, we did not detect any consistent change in errors made by the left hand (no left hand aftereffect in either group), suggesting there was no consistent motor or somatosensory transfer to the left hand.

### Potential neural substrates

Recent years have brought a number of advances in our understanding of the neural substrates of motor adaptation. For example, the early stages of motor adaptation are thought to engage spatial working memory and explicit processes^51^, associated with activations in DLPFC and inferior parietal lobule^52–55^. Late adaptation involves implicit processes to a greater extent^51^. Learning at this point may depend more on the cerebellum^56–59^. Many neuroimaging and patient studies have also suggested that the cerebellum is critical for motor adaptation^5,60–62^. Indeed, non-invasive brain stimulation studies have found that manipulating cerebellar excitability can alter the rate of error reduction in motor adaptation^63–65^. In addition, plasticity in somatosensory cortex is associated with force adaptation^38^; somatosensory training has been found to improve motor adaptation^6,66^.

Several of the brain regions thought to have a role in motor adaptation are also known to have multisensory visuo-proprioceptive properties, suggesting potential neural substrates for our observation of visuo-proprioceptive weighting changes associated with force field adaptation. First, certain parietal regions have been found to respond to both the “seen” and “felt” position of the limb in monkeys^67,68^, suggesting possible involvement in visuo-proprioceptive integration. Second, while the cerebellum has traditionally been classified as a motor structure and its importance in motor adaptation is well known, this structure also has multimodal sensory responses. Indeed, individual cerebellar granule cells have been found to integrate somatosensory, visual, and auditory inputs^69^. In humans, the cerebellum has been implicated in multisensory integration for postural control^70^ and reaction time^71^. Finally, the role in perception of regions historically considered unisensory, such as somatosensory cortex, vs. areas considered multisensory, such as portions of PPC, is far from settled^72^. Such unisensory areas are now known to have multisensory response properties^73^, and likely both modulate, and are modulated by, each other as well as multisensory regions in PPC^72^. In other words, if changes in visuo-proprioceptive integration accompany force field adaptation, as our results suggest, it is plausible that networks containing regions thought to be involved in both processes (PPC, cerebellum, somatosensory cortex) may mediate the interaction.

### No evidence of consistent spatial shifts in proprioceptive or visual estimates

There are several likely reasons we were unable to replicate previous findings of lateral shift in proprioceptive estimate related to force field direction^6,37,38,45,46^. Most importantly, Ostry’s group assesses proprioception with a psychometric 2AFC method that is presumably much more sensitive to small changes than our method of pointing with the left hand. They have reported proprioceptive shifts on the order of 2 mm^46^, and our pointing method is likely too noisy to detect such a small shift. It includes motor and sensory noise from the left (indicator) hand; it is not unusual for a subject’s pointing estimates to extend over a range of 8 cm, as our example subject did for P targets (Fig. 4Ai). Thus, while this method is useful for quickly getting V, P, and VP estimates to compute *w*_*V*_, it is not ideal for detecting millimeter shifts in perception.

Interestingly, Haith *et al*.^28^ did use a pointing method to estimate visual and proprioceptive targets, and did report a shift in both, in the lateral dimension, after adaptation to a leftward lateral force field^28^. There are several methodological differences that could explain why we were not able to replicate this finding: (1) Haith *et al*. used a gradually imposed lateral field, not an abruptly-imposed curl field; (2) V and P estimates were interspersed throughout the force field task, not taken pre and post force field; (3) intermanual transfer of adaptation to the indicator hand was not assessed, nor were results compared with V and P estimates during a null field or rightward force field. In sum, while the Haith *et al*. paper was a smaller experiment in a single group/condition, their gradually-imposed force field and interspersed sensory estimates may be important factors for finding consistent sensory shifts when using a pointing method to obtain V and P estimates.

Unfortunately, the present experiment cannot explain why weighting changes were observed in the sagittal but not lateral dimension, when previous studies have observed proprioceptive shifts in the lateral dimension, and our pointing method did not lend itself to finding small shifts in proprioceptive or visual estimates in either dimension. It is important to note, however, that proprioceptive estimates have not been examined in the sagittal dimension after curl field adaptation using the more sensitive 2AFC method, so it is possible that proprioception does shift in the sagittal dimension. However, spatial recalibration of proprioceptive estimates can be independent of the weighting of vision vs. proprioception^29^, so there may be no connection between proprioceptive shifts and weighting. Another possibility is that weighting is more susceptible to change in the dimension that is most functionally relevant to the subject, which in a straight-ahead-reaching task, could be the sagittal dimension.

## Conclusion

Results of the present study suggest that subjects increase their reliance on vision vs. proprioception when they undergo force field adaptation. This change in visuo-proprioceptive weighting was specific to the sagittal plane, perhaps reflecting the importance of straight-ahead movements in the task design. Force field direction did not play a role, as the effect was comparable for clockwise and counter-clockwise force field exposure. Taken together, these results could indicate that the brain interprets a force field as a somatosensory perturbation and adjusts visuo-proprioceptive integration accordingly.

## Supplementary information

Supplementary Figures

## Data Availability

The datasets generated during and/or analyzed during the current study are available from the corresponding author on request.
