# Supplementary material for: Increase in weighting of vision vs. proprioception associated with force field adaptation
Source: Sci Rep. 2019 Jul 15;9:10167. doi: 10.1038/s41598-019-46625-7 (PMC6629615; doi:10.1038/s41598-019-46625-7)
Supplement: Supplementary file 1 — Supplementary Figures [file 41598_2019_46625_MOESM1_ESM.pdf]

Increase in weighting of vision vs. proprioception associated with force field adaptation

Brandon M. Sexton, Yang Liu, Hannah J. Block*

Indiana University Bloomington, Dept. of Kinesiology & Program in Neuroscience

List of supplementary figures:

S1. CW group mean estimates of V, P, and VP targets

S2. CCW group mean estimates of V, P, and VP targets

S3. Lateral shifts in proprioception (Px)

S4. Lateral shifts in vision (Vx)

S5. Sagittal shifts in proprioception (Py)

S6. Sagittal shifts in vision (Vy)

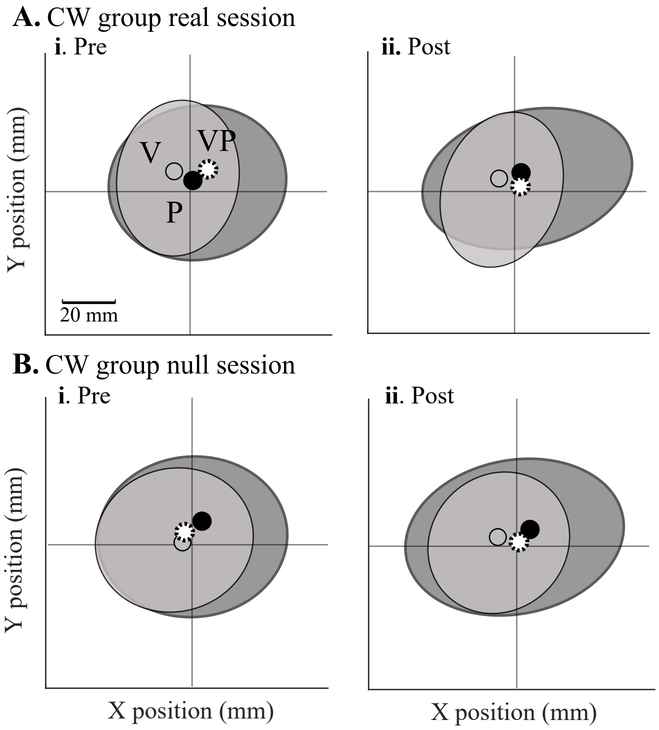

Supplementary Figure S1. CW group mean estimates of V, P, and VP targets. Subject was seated in the direction of the negative y-axis, with the target always at the origin. Group mean estimates of VP, V, and P targets, with standard error ellipses for the latter two target types. The light grey ellipse represents the distribution of subject estimates of the V target, while the dark grey ellipse represents the distribution of subject estimates of the P target. **A.** Real session. i. Pre-reaching task with real CW field. ii. Post-reaching task with real CW field. **B.** Null session. i. Pre-reaching task in null field. ii. Post-reaching task in null field.

Supplementary Figure S2. CCW group mean estimates of V, P, and VP targets. Subject was seated in the direction of the negative y-axis, with the target always at the origin. Group mean estimates of VP, V, and P targets, with standard error ellipses for the latter two target types. The light grey ellipse represents the distribution of subject estimates of the V target, while the dark grey ellipse represents the distribution of subject estimates of the P target. **A.** Real session. i. Pre-reaching task with real CCW field. ii. Post-reaching task with real CCW field. **B.** Null session. i. Pre-reaching task in null field. ii. Post-reaching task in null field.

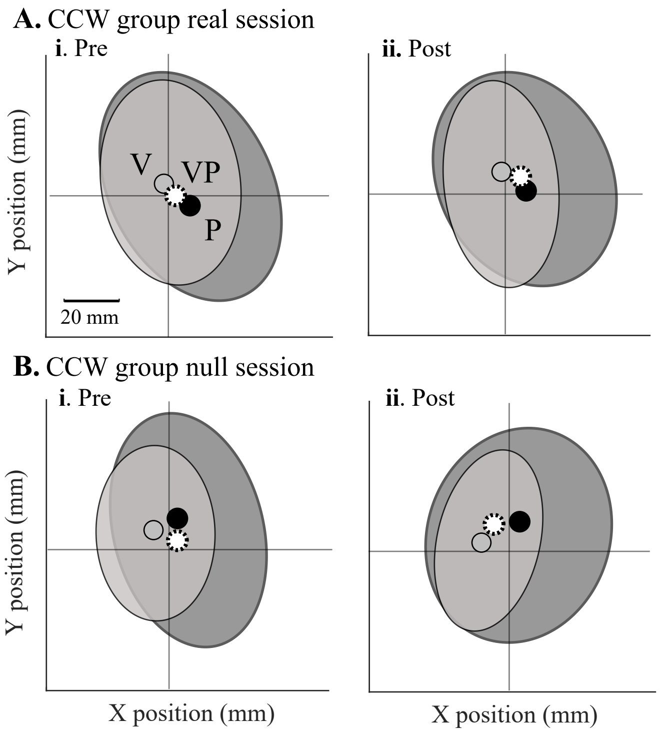

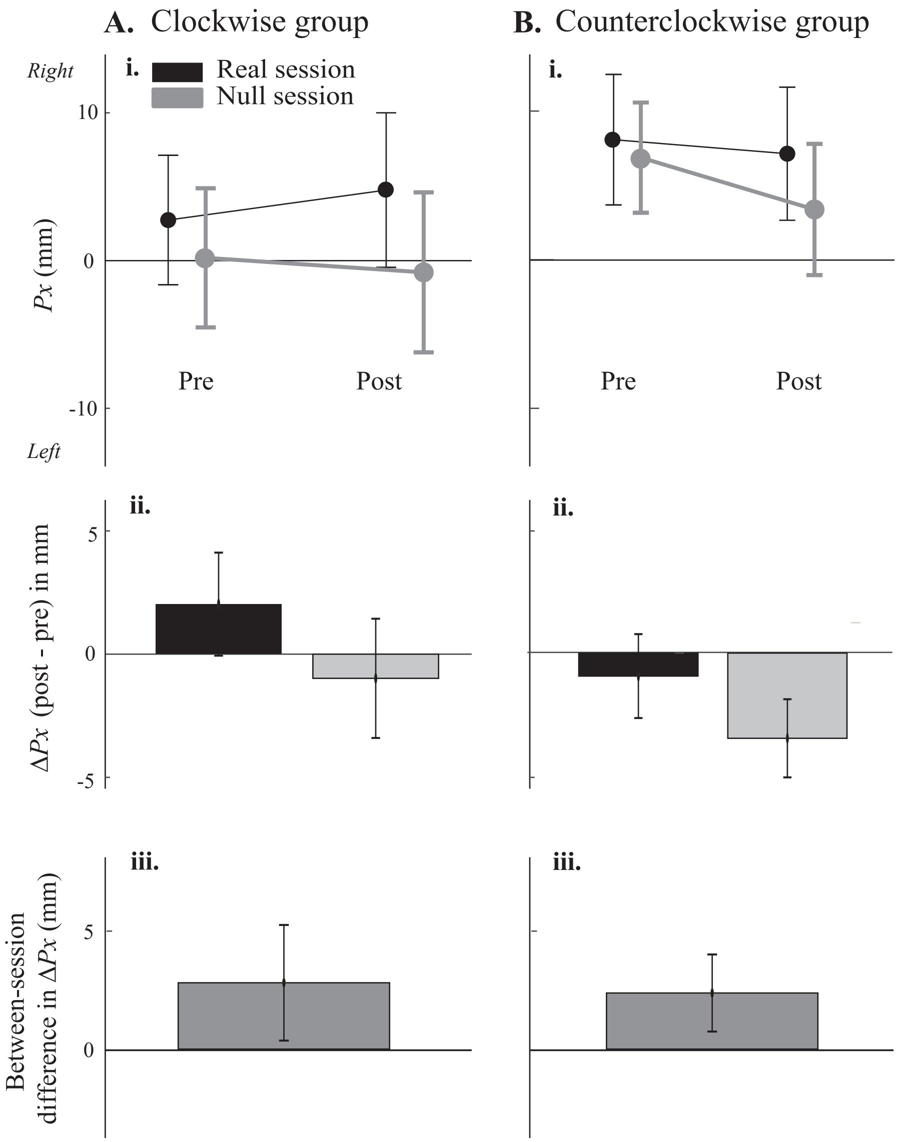

Supplementary Figure S3. Lateral shifts in proprioception (Px) for the clockwise (A) and counterclockwise (B) groups. i. Mean Px pre- and post-adaptation block in the real (black) and null (grey) session. Negative y-axis is to the left of the subject's body midline. ii. Mean within-session change in Px. iii. Mean between-session difference in ΔPx. The absence of interaction effects provides no evidence to suggest that force adaptation affected proprioception in the lateral dimension. All error bars represent standard errors.

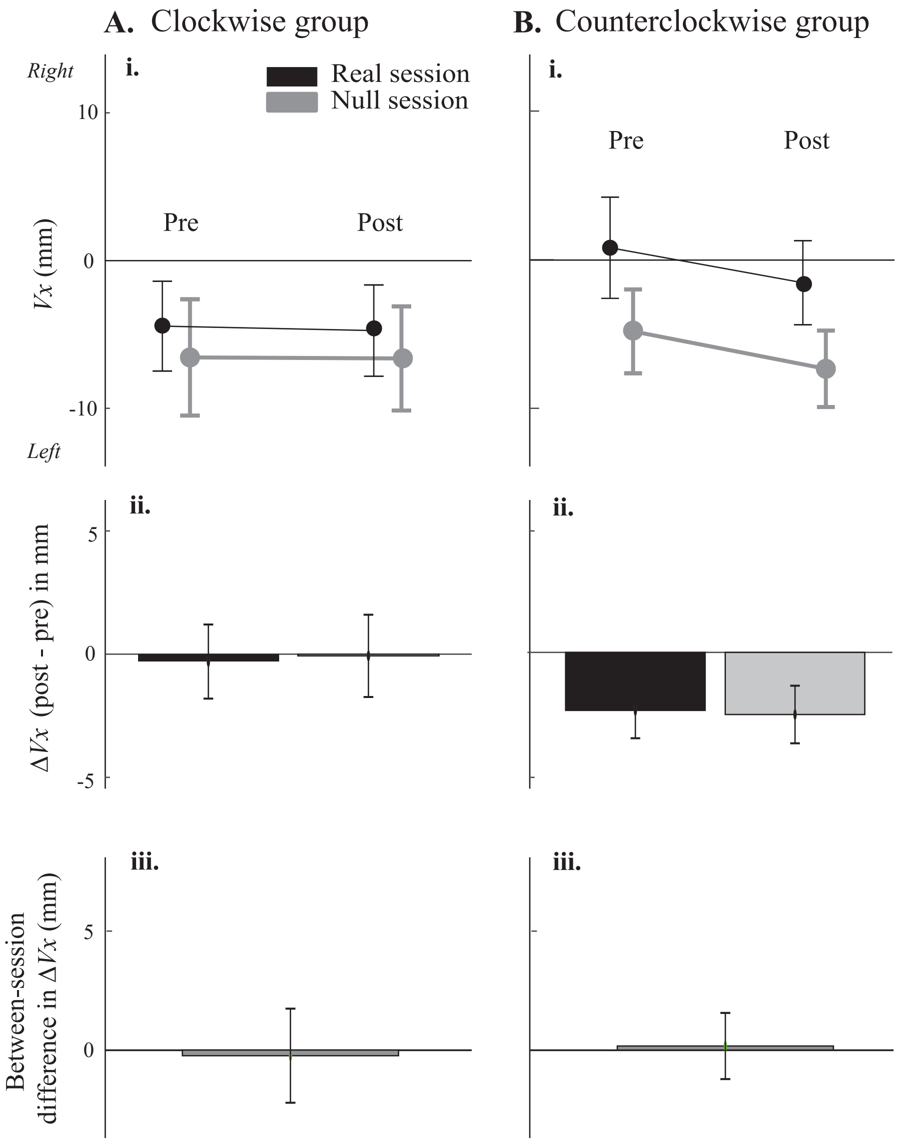

Supplementary Figure S4. Lateral shifts in vision (Vx) for the clockwise (A) and counterclockwise (B) groups. i. Mean Vx pre- and post-adaptation block in the real (black) and null (grey) session. Negative y-axis is to the left of the subject's body midline. ii. Mean within-session change in Vx. iii. Mean between-session difference in ΔVx. The absence of interaction effects provides no evidence to suggest that force adaptation affected vision in the lateral dimension. All error bars represent standard errors.

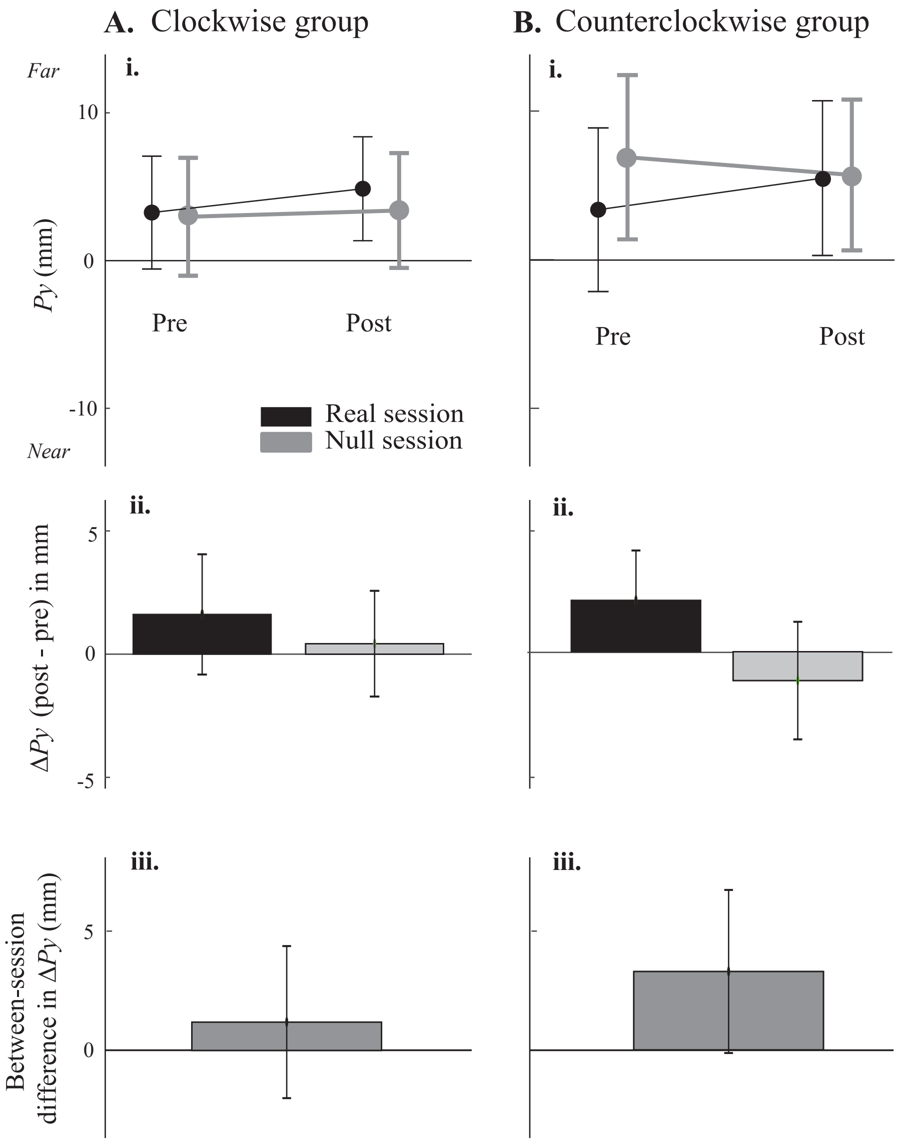

Supplementary Figure S5. Sagittal shifts in proprioception (Py) for the clockwise (A) and counterclockwise (B) groups. i. Mean Py pre- and post-adaptation block in the real (black) and null (grey) session. Negative y-axis is closer to the subject. ii. Mean within-session change in Py. iii. Mean between-session difference in ΔPy. The absence of interaction effects provides no evidence to suggest that force adaptation affected proprioception in the sagittal dimension. All error bars represent standard errors.

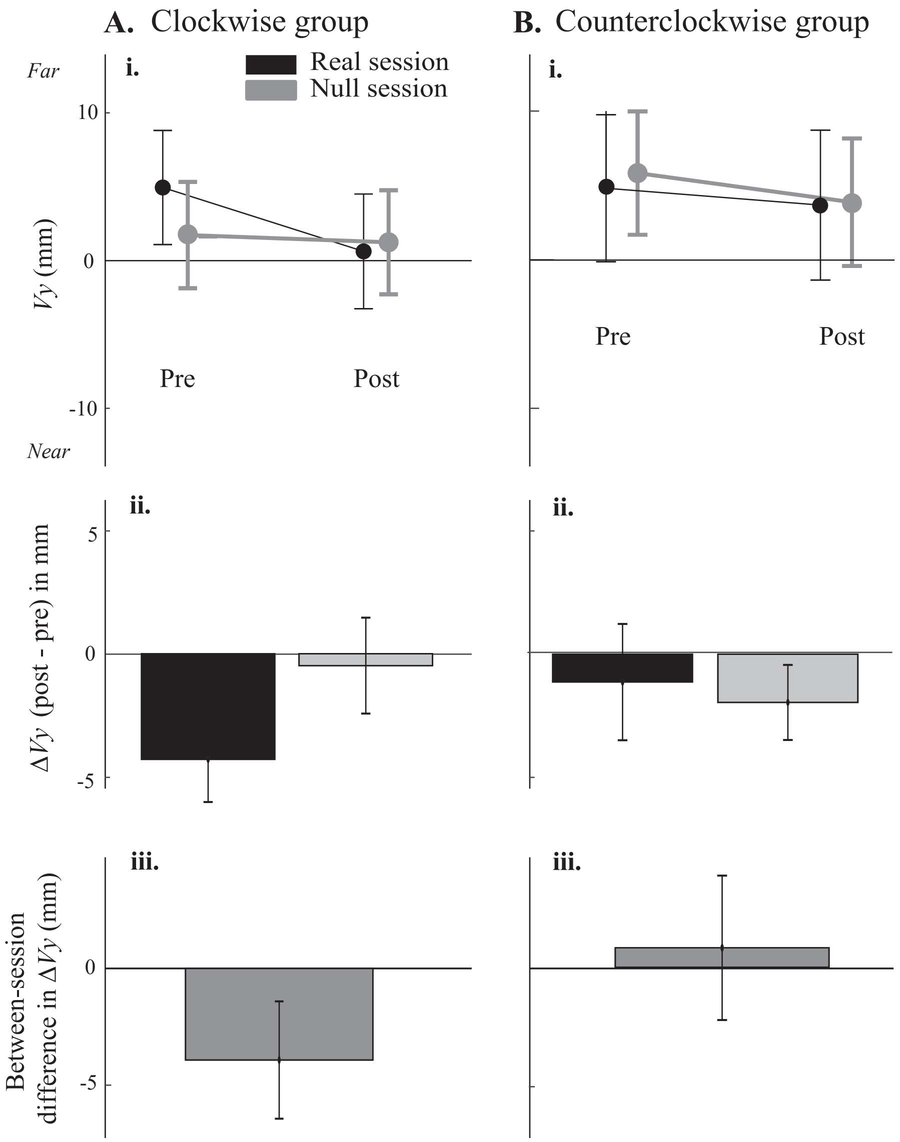

Supplementary Figure S6. Sagittal shifts in proprioception (Vy) for the clockwise (A) and counterclockwise (B) groups. i. Mean Vy pre- and post-adaptation block in the real (black) and null (grey) session. Negative y-axis is closer to the subject. ii. Mean within-session change in Vy. iii. Mean between-session difference in ΔVy. The absence of interaction effects provides no evidence to suggest that force adaptation affected vision in the sagittal dimension. All error bars represent standard errors.
